# Multiple Myeloma: A Forgotten Imitator of Anti-Neutrophil Cytoplasmic Antibody (ANCA) Vasculitis

**DOI:** 10.7759/cureus.77647

**Published:** 2025-01-19

**Authors:** Adriana M Trejos Tenorio, Carlos A Regino, Alex J Imbachí-Salamanca, Harold Dávila, Carlos H Muñoz Vahos

**Affiliations:** 1 Internal Medicine, Hospital Quirónsalud Torrevieja, Torrevieja, ESP; 2 Hematology, National University of Colombia, Bogotá, COL; 3 Rheumatology, Hospital Universitario San José, Popayán, COL; 4 Pathology, Laboratorio de Patologia y Citologia (LAPACI), Medellín, COL; 5 Rheumatology, University of Antioquia, Hospital San Vicente Fundación, Medellín, COL

**Keywords:** anca associated vasculitis, cutaneous manifestations of multiple myeloma, lekocytoclastic vasculitis, palpable purpura, skin ulcer

## Abstract

Leukocytoclastic vasculitis is a neutrophilic angiitis with diverse etiologies, including both inflammatory and neoplastic conditions, such as anti-neutrophil cytoplasmic antibody-associated vasculitis (AAV) and multiple myeloma (MM). Skin involvement in MM is rare, occurring in less than 1% of cases. We present a case of a patient with retiform purpura and peripheral neuropathy in the lower limbs, secondary to IgG lambda MM, which mimicked AAV. Skin manifestations in MM can appear as the initial symptom or at any stage of the disease. This case highlights the critical role of protein electrophoresis in the initial evaluation of patients with systemic inflammatory conditions.

## Introduction

Palpable purpura is the most common clinical manifestation of small vessel vasculitis, with histological findings consistent with leukocytoclastic vasculitis (LV), a neutrophilic angiitis characterized by fibrinoid necrosis of the vessel walls. A variety of etiologies are associated with this condition, including (1) systemic infections (e.g., HIV, hepatitis B, hepatitis C, and syphilis); (2) drugs (e.g., cocaine-levamisole, antibiotics); (3) systemic inflammatory conditions (e.g., anti-neutrophil cytoplasmic antibody (ANCA)-associated vasculitis (AAV), IgA vasculitis, cryoglobulinemic vasculitis, and urticarial vasculitis); and (4) solid tumors and hematologic malignancies [1,2].

Skin involvement in multiple myeloma (MM) is rare, occurring in less than 1% of cases, and may present as either an initial symptom or at any stage of the disease [1].

This article aims to present a case of MM mimicking AAV and emphasize the role of protein electrophoresis in the differential diagnosis of patients with systemic inflammatory conditions.

## Case presentation

**Figure 1:**
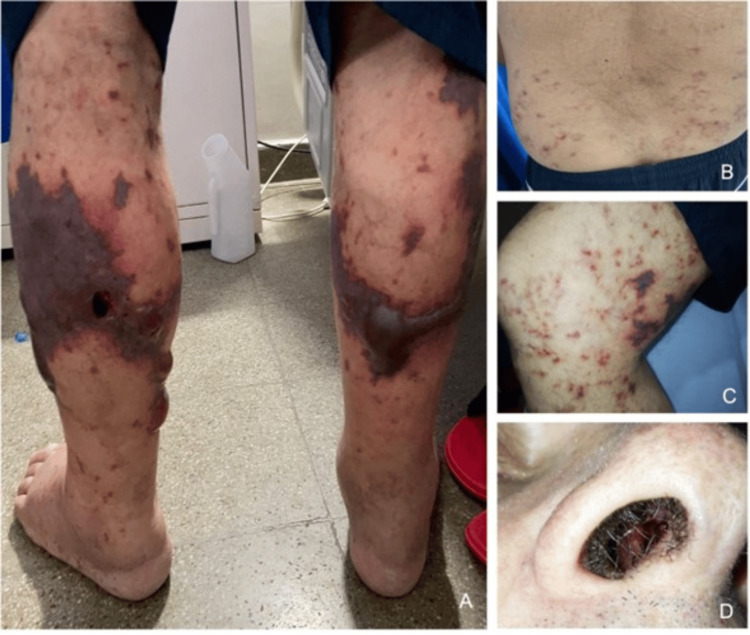
Mucocutaneous findings (A) Retiform purpura and hemorrhagic bullae on the legs. (B) Retiform purpura in the lumbar region. (C) Retiform purpura on the thighs. (D) Nasal crusts with active epistaxis.

A 68-year-old man with a history of hypertension and cigarette smoking presented to our emergency department with a three-month history of palpable purpura, paresthesia, allodynia, progressive lower extremity soft tissue edema, pitting edema, and a 5-kg weight loss. Two weeks before admission, he had experienced epistaxis and hemorrhagic bullae on his lower limbs. Physical examination revealed hematic crusts in the nostrils without septal perforation and retiform purpura (Figure 1).

**Figure 2:**
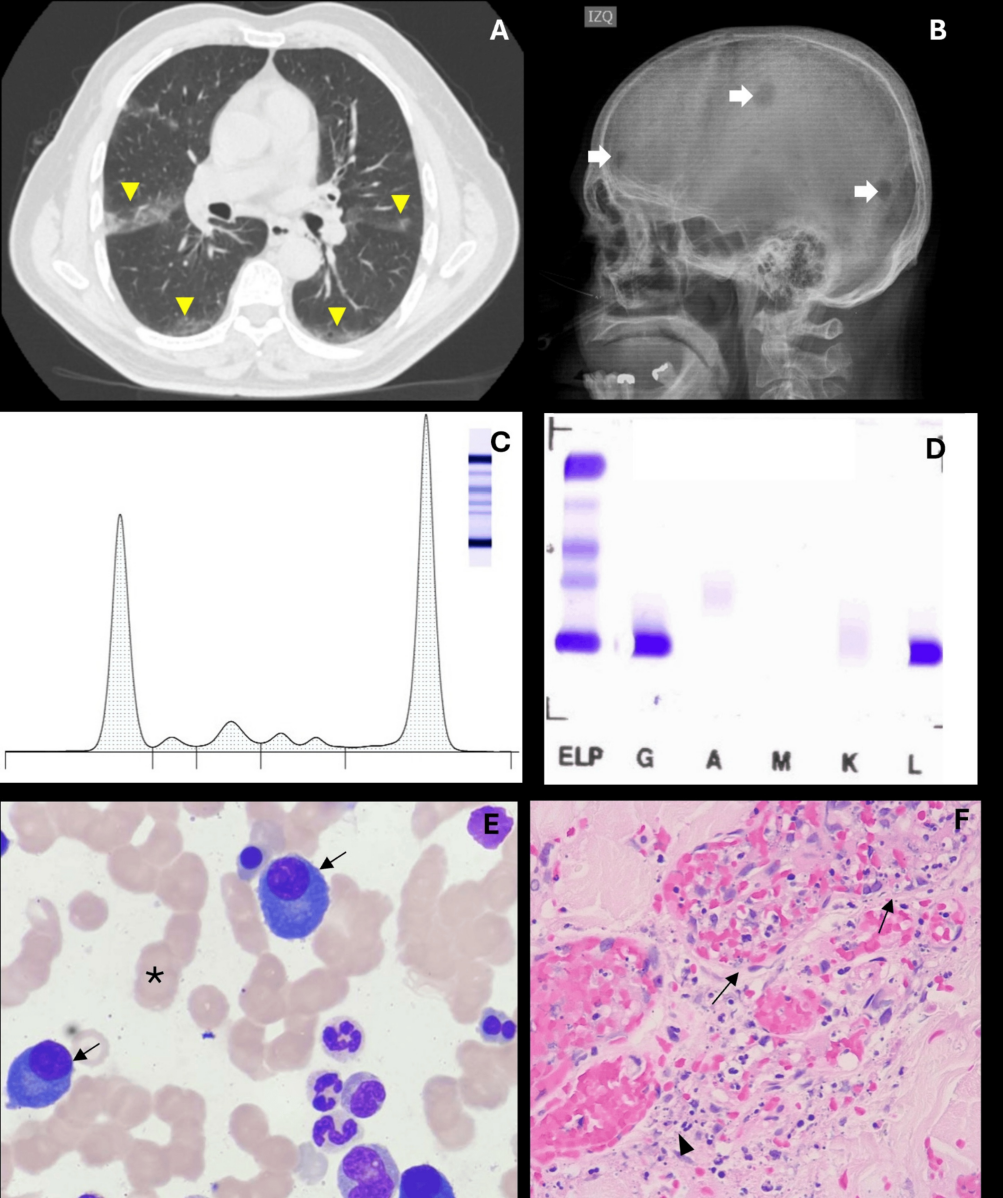
Clinical, immunological, and bone marrow findings (A) Chest tomography revealing ground-glass opacities (yellow triangles). (B) Lytic lesions in the skull (white arrows). (C) Serum protein electrophoresis showing a monoclonal gamma peak. (D) Serum immunofixation positive for IgG-lambda. (E) Bone marrow biopsy displaying Rouleaux formation (asterisk) and 30% atypical plasma cells (arrows). (F) Skin biopsy (40× magnification) demonstrating a perivascular mixed inflammatory infiltrate of lymphocytes, plasma cells, and neutrophils (arrows), nuclear dust (triangle), and foci of fibrinoid necrosis in the vessel wall, consistent with LV. LV, leukocytoclastic vasculitis

Laboratory findings revealed hemoglobin: 10.6 g/dL, MCV: 98.2 fL, and a normal CBC and differential. Erythrocyte sedimentation rate was 120 mm/h, CRP was 6.07 mg/dL, albumin was 2.8 g/dL, calcium was 8.9 mg/dL, creatinine was 1.9 mg/dL, glomerular filtration rate was 38 mL/min/1.73 m², BUN was 57.9 mg/dL, and 24-hour urine protein was 1,151 mg. Infectious workup, including HIV, hepatitis B and C viruses, and urine cocaine, was negative. Immunologic tests, including antinuclear antibodies by indirect immunofluorescence, anti-DNA antibodies, extractable nuclear antigens, and paired cryoglobulins, were also negative. Only complement studies revealed moderate C4 hypocomplementemia (Table 1). Chest CT showed peripheral ground-glass opacities (Figure 2A), and the skin biopsy confirmed LV (Figure 2F).

**Table 1:** Blood test results Ag-VHB, hepatitis B surface antigen; Ab-VHC, hepatitis C antibodies; ANA, antinuclear antibodies; anti-DNA, anti-DNA antibodies; BUN, blood urea nitrogen; C3, complement component 3; C4, complement component 4; ENA, extractable nuclear antigens; ESR, erythrocyte sedimentation rate; GFR, glomerular filtration rate; MCV, mean corpuscular volume; MPO, myeloperoxidase antibody; PR3, proteinase 3

Test	Result	Unit	Reference range
Hemoglobin	10.6	g/dL	12-16
Hematocrit	32.7	%	38-48
MCV	98.2	fL	86-96
Leukocytes	8.980	× 10³/µL	4.5-11.0
Platelets	213	× 10³/µL	150-450
ESR	120	mm/h	0-20
CRP	6.07	mg/dL	0.01-1.00
Albumin	2.85	g/dL	3.4-4.8
Calcium	8.9	mg/dL	8.8-10.3
Creatinine	1.9	mg/dL	0.5-0.8
GFR	38	mL/min/1.73m^²^	90-120
BUN	57.9	mg/dL	6-24
24-hour urine protein	1151	mg/24h	0-150
HIV	0	index	0
Ag-VHB	<0.1	index	0
Ab-VHC	0	index	0
ANA	Negative	index	<1:80
ENA total	<20	UE/mL	<20
Anti-DNA	0	U/mL	Negative
C3	113	mg/dL	90-170
C4	4.8	mg/dL	12-36
Anti-MPO	1.1	U	<20
Anti-PR3	1.2	U	<20

Granulomatosis with polyangiitis was initially suspected, and the patient received pulse steroid therapy with 500 mg of IV methylprednisolone for three days. ANCAs by enzyme-linked immunosorbent assay were negative. Protein electrophoresis revealed a monoclonal IgG peak (5,286.77 g/dL), and immunofixation demonstrated an IgG lambda monoclonal band. Bone marrow biopsy confirmed the diagnosis of MM (Figure 2). Treatment with daratumumab and bortezomib-cyclophosphamide-dexamethasone was initiated. The patient had an excellent clinical response, with remission of skin ulcers.

## Discussion

This patient presented with clinical features suggestive of AAV, including peripheral neuropathy and palpable purpura. However, these findings ultimately revealed an unusual manifestation of MM. This case emphasizes the importance of a comprehensive differential diagnosis in patients with suspected small-vessel vasculitis. Considering toxic, infectious, inflammatory, and oncologic etiologies, such as MM, is crucial.

LV is a heterogeneous condition characterized by neutrophilic inflammation of small blood vessels. It can be associated with various underlying conditions, including hematological malignancies, leading to clinical presentations that mimic primary vasculitides, particularly those linked to ANCA.

Skin involvement, including palpable purpura and ulcers, is rare in MM but can occur as an initial symptom or at any stage of the disease. An observational study documented LV in eight out of 2,357 MM patients, presenting as palpable purpura in the lower extremities, with or without systemic symptoms [1]. Other cutaneous manifestations of MM include plasmacytomas, pyoderma gangrenosum, bullous diseases, and cutaneous amyloidosis [3,4]. Clinical suspicion of MM based solely on cutaneous findings remains infrequent.

To date, including our reported case, 25 cases of MM-associated LV have been described in the literature. The male-to-female ratio is similar, with IgG myeloma being more frequent. Cutaneous manifestations can precede the diagnosis of MM, serve as the initial presenting symptom, or, less commonly, occur during treatment with thalidomide (Table 2).

**Table 2:** Cases reported in the literature of mucocutaneous findings in MM LV, leukocytoclastic vasculitis; MM, multiple myeloma

Reference	Age	Sex	Presentation	Ig	Comment	Outcome
McMillen et al. (1986) [5]	58	F	Diagnosis	IgA kappa	In relapses: LV	Remission
Bayer-Garner and Smoller (2003) [1]	58	M		IgA lambda		Not available
	67	F		Not available		Not available
	42	F		IgA kappa		Not available
	67	M		IgG kappa		Not available
	57	M		IgG kappa		Not available
	73	M		IgG kappa		Not available
	66	M		IgG lambda		Not available
	41	F		Not available		Not available
Witzens et al. (2004) [6]	62	M	During treatment	IgG kappa	Associated with thalidomide	Improvement with steroids and discontinuation of thalidomide
Cem Ar et al. (2005) [7]	51	F	At diagnosis	IgG kappa	Cryoglobulins type I	Improvement with dexamethasone and thalidomide
Min et al. (2006) [8]	55	M	During treatment	IgA kappa	Bortezomib	Self-limited
Kembre et al. (2006) [9]	52	M	Diagnosis	Not available	Necrotic ulcers	Not available
	42	M	Diagnosis	IgG	Purpura retiformis - cryoglobulins	Died of respiratory failure
Yildirim et al. (2007) [10]	58	M	During treatment	Kappa light chains	Associated with thalidomide	Remission
Jain et al. (2009) [11]	53	M	Diagnosis	IgG kappa	Necrotic ulcers	Remission
Fritsch and Azambuja (2010) [12]	75	F	Diagnosis	IgG kappa	Cryoglobulinemia	Relapse with the same injuries
Peterlin et al. (2011) [13]	58	F	Diagnosis	IgA lambda	Rapid progression	Remission
Çağırgan et al. (2012) [14]	35	M	Diagnosis	IgG kappa	Necrotic ulcers and pulmonary calcinosis	Partial remission with QT
Abouzaid et al. (2013) [15]	48	F	Diagnosis	IgA lambda	Purple	Remission
Ichiyama et al. (2017) [16]	76	M	During treatment	IgA kappa	Associated with thalidomide and tissue eosinophilia	Improvement with suspension
Oka et al. (2018) [17]	85	F	Diagnosis	IgG kappa	Eosinophilia	Partial remission
Mejía-Zuluaga et al. (2020) [18]	46	F	Diagnosis	Kappa	Sd blue finger	Remission
Runge et al. (2021) [19]	62	F	Diagnosis	IgG lambda	Necrotic ulcers and cryoglobulinemia	Remission
Maciel et al. (2024) [20]	76	F	Diagnosis	IgG kappa	Necrotic ulcers and peripheral neuropathy	High-intensity laser for ulcers
Our case	68	M	Diagnosis	IgG lambda	Necrotic ulcers and peripheral neuropathy	Remission

This case underscores the critical role of protein electrophoresis in the initial evaluation of patients with systemic inflammatory conditions. The presence of a monoclonal protein peak serves as a key diagnostic clue, prompting consideration of monoclonal gammopathies as the underlying etiology.

Differential diagnosis from small-vessel vasculitis is essential. In this case, C4 hypocomplementemia, an unusual finding in primary AAV, raised concerns about an alternative diagnosis.

## Conclusions

Palpable purpura is a common clinical finding in various conditions, including infections, drug reactions, systemic inflammatory diseases, and malignancy. It is typically associated with systemic vasculitides affecting small vessels, such as AAV. While skin involvement as the first clinical sign in MM is rare, it has been reported at any stage of the disease. A systematic approach, including serum electrophoresis, is crucial for narrowing the differential diagnosis in inflammatory multisystemic conditions.
